# 2010-2019年甘肃省肿瘤登记地区肺癌发病的流行病学特征分析

**DOI:** 10.3779/j.issn.1009-3419.2024.102.05

**Published:** 2024-02-20

**Authors:** Zhaoxia LU, Jixiong MA, Juhong MA, Hong ZHOU, Juanjuan XUE, Gaoheng DING, Yindi WANG, Zhen LV, Yuqin LIU, Ben WANG, Lili CHEN

**Affiliations:** ^1^730900 白银，白银市疾病预防控制中心（鲁朝霞，马骥雄，马菊红，周红，薛娟娟，王犇）; ^1^Baiyin Center for Disease Control and Prevention, Baiyin 730900, China; ^2^730050 兰州，甘肃省肿瘤医院（丁高恒，刘玉琴，陈莉莉）; ^2^Gansu Cancer Hospital, Lanzhou 730050, China; ^3^730050 兰州，甘肃中医药大学公共卫生学院（王引弟，吕珍）; ^3^Public Health College of Gansu University of Chinese Medicine, Lanzhou 730050, China

**Keywords:** 肺肿瘤, 发病特征, 变化趋势, Lung neoplasms, Incidence characteristics, Change trend

## Abstract

**背景与目的** 肺癌是中国发病率最高、疾病负担最重的恶性肿瘤。近年来，肺癌呈高发趋势，严重影响人群健康。本文通过分析甘肃省肿瘤登记地区2019年肺癌发病特征及2010-2019年发病率趋势，为甘肃省肺癌防控策略制定提供参考依据。** 方法** 通过分析甘肃省肿瘤登记地区2019年肺癌发病个案，计算发病率、中标发病率、世标发病率等相关指标；采用Joinpoint计算年度变化百分比（annual percent change, APC），进行趋势分析。** 结果** 2019年甘肃省共报告肺癌新发病例3757例，占所有新发恶性肿瘤的14.96%，肺癌发病率、中标率、世标率分别为40.52/10万、30.91/10万和25.86/10万，0-74岁累积率、35-64岁截缩率分别为3.23%和40.03/10万。肺癌发病随着年龄增长而上升，在≥40岁组高发，男、女性人群分别在≥75岁组、≥80岁组达到发病高峰。2010-2019年甘肃省肿瘤登记地区肺癌粗发病率总体呈上升趋势，且上升速度较快，APC为5.39%（P<0.05）；分别按照性别、城乡进行统计，各人群肺癌发病率均呈上升趋势，男性、女性、城市、农村人群APC分别为4.98%、6.39%、6.26%和4.64%（P均<0.05）。按照年龄组肺癌发病率进行趋势分析，仅≥65岁组人群肺癌发病以年均4.15%的速度上升（P<0.05）。** 结论** 2010-2019年甘肃省肿瘤登记地区肺癌发病率呈现逐年上升趋势，不同性别、地区及年龄组人群肺癌发病存在差异，应针对肺癌发病重点人群开展综合防控工作。

## Abstract (en)

**Background and objective** Lung cancer is the malignant tumor with the highest incidence rate and the heaviest disease burden in China. In recent years, lung cancer has shown a high incidence trend, seriously affecting the health of the population. In this paper, we analyze the characteristics of lung cancer incidence in 2019 and the trend of incidence rate from 2010-2019 in the tumor registration area of Gansu province, in order to provide a reference basis for the development of lung cancer prevention and control strategies in Gansu province. **Methods** By analyzing the cases of lung cancer incidence in the tumor registration area of Gansu province in 2019, we calculated the incidence rate, medium incidence rate, world incidence rate and other related indexes; we used Joinpoint to calculate the annual percentage change (APC) for trend analysis. **Results** In 2019, a total of 3757 new cases of lung cancer were reported in Gansu province, accounting for 14.96% of all new malignant tumors. The incidence rate, medium incidence rate and world incidence rate and world rate of lung cancer were 40.52/10^5^, 25.78/10^5^, 25.86/10^5^; and the cumulative rate of 0-74 years old, and the truncation rate of 35-64 years old were 3.23%, 40.03/10^5^, respectively. The incidence of lung cancer rises with age, and is high in the age group of 40 years and above, and the incidence peaks in the male and female populations in the group of 75 years and above, and the group of 80 years and above, respectively. The crude incidence rate of lung cancer in the tumor registration area of Gansu province from 2010-2019 showed an overall increasing trend, and the rate of increase was relatively fast, with an APC 5.39% (P<0.05); Separately, according to gender, urban and rural areas, the incidence of lung cancer in all populations showed an increasing trend, and the APC of male, female, urban and rural populations were 4.98%, 6.39%, 6.26%, and 4.64%, respectively (all P<0.05). According to the trend analysis of lung cancer incidence rate by age group, only lung cancer incidence in the age group of 65 years and above increased at an annual average rate of 4.15% (P<0.05). **Conclusion** The incidence rate of lung cancer in the tumor registration area of Gansu province from 2010 to 2019 shows a rising trend year by year, and there are differences in the incidence of lung cancer in people of different genders, regions and age groups, so comprehensive prevention and control work should be carried out for the key populations of lung cancer incidence.

肺癌是中国发病率最高、癌症负担最重的恶性肿瘤^[1,2]^，也是世界各国发病率较高的恶性肿瘤之一^[3]^。全球癌症报告数据（GLOBOCAN）^[3]^显示，2020年全球新发肺癌220.7万例，占全部新发恶性肿瘤的11.4%。近年来，随着中国人口老龄化的不断加剧及肺癌筛查项目在全国范围内的大力开展，中国肺癌的发病呈现缓慢上升的趋势^[3⇓-5]^，中国2022年肺癌新发病例数占所有恶性肿瘤新发病例数的18.06%，居恶性肿瘤发病第1位^[6]^。肺癌早期大多无明显症状，多数患者出现临床症状就诊时基本已处于晚期，晚期患者的5年生存率仅为20%左右^[7]^。因此，明确肺癌流行特征并大力推广和实施肺癌早诊早治项目对于有效降低肺癌发病并提高肺癌患者5年生存率具有重要意义^[8]^。本文对甘肃省2010-2019年肺癌发病情况进行分析，旨在为进一步优化和完善甘肃省肺癌防控工作举措、有效降低肺癌发病提供科学依据与数据支持。

## 1 资料与方法

### 1.1 数据来源

甘肃省2010-2019年肺癌发病数据来源于甘肃省癌症中心。甘肃省癌症中心挂靠在甘肃省肿瘤医院，承担甘肃省各肿瘤登记处肿瘤数据的收集、审核和汇总分析工作。按照《国家肿瘤年报》数据收录标准，2019年甘肃省共有23个肿瘤登记点（12个城市肿瘤登记点、11个农村肿瘤登记点）的数据质量达到了《国家肿瘤年报》收录标准。本研究将2010-2019年甘肃省达到《国家肿瘤年报》数据收录标准的全部数据纳入分析。所有病例均按照第十版《国际疾病分类标准》进行编码，将编码为C33-C34的所有个案纳入分析。人口资料来源于公安局和统计部门。标准人口对照选取2010年全国普查人口和Segi世界标准人口。

### 1.2 质量评价

统一采用国际通用的肿瘤数据质量评价标准对数据的完整性、可靠性进行质量评价，2010-2019年甘肃省肺癌病例中由病理组织学诊断（proportion of morphological verification, MV%）的患者在46.43%-68.44%，平均为57.45%，城市地区为60.67%，农村地区为49.39%；各年份仅有死亡医学证明书的比例（percentage of cancer cases identified with death certification only, DCO%）平均为1.39%，城市地区为0.56%，农村地区为3.56%，均低于10%；诊断部位不明比例（proportion of unknown basis of diagnosis, UB%）平均为0.22%，其中城市地区为0.18%，农村地区为0.34%，均低于5%；死亡/发病比（mortality to morbidity ratio, M/I）在0.57-0.79，平均为0.67，城市地区为0.65，农村地区为0.71；各数据质量评价指标提示2010-2019年甘肃省肿瘤登记地区肺癌发病数据具有较好的可靠性和完整性。

### 1.3 统计学处理

应用SAS 9.4和Excel 2019软件进行统计分析，计算不同时间、不同性别、不同地区肺癌发病率、中国人口标化发病率（简称中标率）（age-standardized incidence rates by 2010’s Chinese standard population, ASIRC）、世界人口标化发病率（简称世标率）（age-standardized incidence rates by World Segi’s population, ASIRW）、0-74岁累积率（cumulative incidence, CI）、35-64岁截缩率（truncation rate incidence, TRI）；采用JoinPoint 5.2软件计算年变化百分率（annual percentage change, APC）进行变化趋势描述和统计检验，检验水准α=0.05。

## 2 结果

### 2.1 2019年肺癌发病总体情况

2019年甘肃省共报告肺癌新发病例3757例，占所有新发恶性肿瘤的14.96%，其中男性2494例（占66.38%），女性1263例（占33.62%）；城市地区2541例（占67.63%），农村地区1216例（占32.37%）。2019年甘肃省肺癌粗发病率、中标率、世标率分别为40.52/10万、30.91/10万和25.86/10万；男性粗发病率、中标率、世标率分别为52.83/10万、42.87/10万和34.83/10万，高于女性的27.75/10万、21.22/10万和17.07/10万，城市地区的发病率、中标率、世标率分别为44.39/10万、33.32/10万和27.15/10万，均高于农村地区的34.28/10万、29.42/10万和23.58/10万；0-74岁累积率、35-64岁截缩率分别为3.23%和40.03/10万，其中男性为4.45%、52.07/10万，均高于女性的2.02%、27.77/10万，城市地区为3.44%、41.76/10万，均高于农村地区的2.86%、36.97/10万。见表1。

**表1:** 2019年甘肃省肿瘤登记地区肺癌发病情况

Area	Gender	Case (n)	Crude rate (/10^5^)	ASIRC (/10^5^)	ASIRW (/10^5^)	CI (%)	TRI (/10^5^)
Total	Total	3757	40.52	30.91	25.86	3.23	40.03
	Male	2494 (66.38%)	52.83	42.87	34.83	4.45	52.07
	Female	1263 (33.62%)	27.75	21.22	17.07	2.02	27.77
Urban	Total	2541	44.39	33.32	27.15	3.44	41.76
	Male	1759 (69.22%)	60.60	46.57	38.01	4.93	55.53
	Female	782 (30.78%)	27.71	20.43	16.52	1.97	27.88
Rural	Total	1216	34.28	29.42	23.58	2.86	36.97
	Male	735 (60.44%)	40.43	36.01	29.01	3.61	45.92
	Female	481 (39.56%)	27.81	22.88	18.18	2.12	27.68

ASIRC: age-standardized incidence rates by 2010’s Chinese standard population; ASIRW: age-standardized incidence rates by World Segi’s population; CI: cumulative incidence; TRI: truncation rate incidence.

### 2.2 2019年肺癌年龄别发病情况

2019年甘肃省肺癌发病率随着年龄的增长呈上升趋势，在40岁之前，发病处于较低水平，40岁开始呈缓慢增长，55岁开始快速上升。男性在≥75岁组达到发病高峰，城市地区和农村地区发病率分别为340.84/10万和246.15/10万，≥80岁年龄组发病率有所下降；女性人群在≥80岁组达到发病高峰，城市地区和农村地区发病率分别为148.03/10万和216.49/10万，≥85岁年龄组发病率呈下降趋势；≥50岁组及以后男性各年龄组肺癌发病率明显高于女性。见图1。

**图1:**
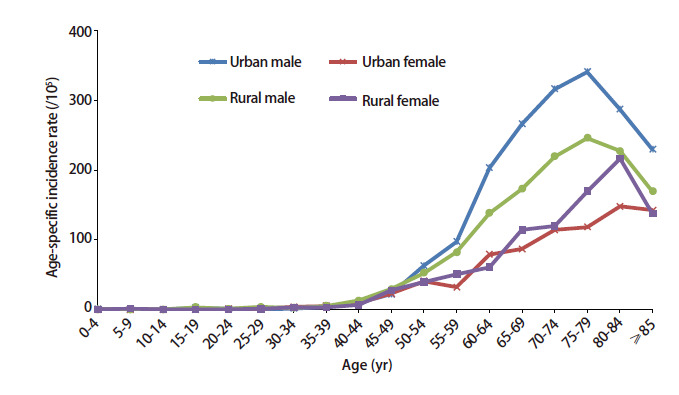
2019年甘肃省肿瘤登记区肺癌年龄别发病率

### 2.3 2019年肺癌发病地区分布

2019年甘肃省肿瘤登记地区12个城市肿瘤登记点中肺癌发病率最高的为兰州市西固区，为77.37/10万，占所有恶性肿瘤发病的25.67%，最低的是酒泉市敦煌市，为20.82/10万，占9.52%；11个农村点中发病率最高的为庆阳市庆城县，为45.67/10万，占20.26%，最低的是武威市天祝县，为26.91/10万，占13.61%。见表2。

**表2:** 2019年甘肃省不同地区肺癌发病情况

Order	Urban							Rural					
	Area	Case (n)	Ratio (%)	Crude rate (/10^5^)	ASIRC (/10^5^)	ASIRW (/10^5^)		Area	Case (n)	Ratio (%)	Crude rate (/10^5^)	ASIRC (/10^5^)	ASIRW (/10^5^)
1	Xigu	248	25.67	77.37	34.93	35.90		Qingcheng	125	20.26	45.67	31.35	31.57
2	Chenggua	574	16.03	59.99	30.72	30.53		Lintan	65	17.15	45.62	38.56	37.24
3	Qilihe	308	19.18	52.79	42.12	44.08		Gaotai	63	15.29	42.94	26.57	27.12
4	Pingchuan	99	16.72	46.62	32.75	32.56		Minqin	101	12.58	41.81	23.76	23.66
5	Baiyin	148	15.38	46.55	24.32	24.78		Jingtai	98	15.38	41.01	38.23	38.47
6	Anning	100	16.45	45.56	26.08	25.47		Jingning	167	12.60	34.65	28.02	27.77
7	Qinzhou	258	18.79	38.74	34.40	35.38		Huining	184	11.61	33.83	22.67	22.31
8	Maiji	206	18.85	37.26	22.62	23.21		Jingyuan	147	14.70	31.40	23.02	23.10
9	Ganzhou	179	11.78	34.52	22.96	22.51		Lintao	166	14.13	30.49	21.31	21.23
10	Liangzhou	317	10.71	29.14	19.04	18.95		Gulang	78	8.44	29.53	17.17	16.74
11	Honggu	41	14.86	28.92	19.64	19.64		Tianzhu	55	13.61	26.91	16.77	16.88
12	Dunhuang	30	9.52	20.82	17.79	17.26							

### 2.4 2010-2019年肺癌发病率变化趋势

2010-2019年甘肃省肿瘤登记地区肺癌发病率以年均5.39%的速度上升（P<0.05），中标率、世标率整体呈平稳趋势（中标率：APC为1.09%，世标率：APC为2.26%，P均>0.05）；男性肺癌发病率APC为4.98%，女性APC为6.39%，城市地区APC为6.26%，农村地区APC为4.64%（P均<0.05），除女性人群肺癌发病中标率呈上升趋势（中标率：APC为3.92%，P<0.05）外，其余人群中标率、世标率均呈平稳趋势（P均>0.05）。见表3、图2。

**表3:** 2010-2019年甘肃省肿瘤登记地区肺癌发病趋势（/10^5^）

Year	Male				Female				Urban				Rural				Total		
	Crude rate	ASIRC	ASIRW		Crude rate	ASIRC	ASIRW		Crude rate	ASIRC	ASIRW		Crude rate	ASIRC	ASIRW		Crude rate	ASIRC	ASIRW
2010	29.98	35.28	28.20		15.05	16.11	12.81		22.70	25.09	20.05		23.35	21.97	21.90		22.86	25.71	20.52
2011	34.53	37.81	30.20		17.57	17.92	14.37		24.93	25.56	20.45		30.19	28.26	28.22		26.28	27.85	22.28
2012	30.43	35.59	28.10		15.88	16.77	13.70		22.13	22.89	18.45		27.05	32.32	31.32		23.37	26.13	20.86
2013	36.95	40.04	32.19		22.23	22.13	17.77		27.11	27.77	22.47		37.95	34.01	33.38		29.82	31.09	25.01
2014	36.95	40.66	33.93		18.93	18.90	14.66		26.95	26.34	21.76		31.87	36.10	35.61		28.17	29.68	24.24
2015	36.87	38.05	31.08		18.64	17.64	13.93		27.60	26.53	21.60		29.25	26.44	25.83		28.01	27.73	22.42
2016	36.34	37.91	30.19		19.18	18.12	14.67		27.80	26.26	21.22		28.41	27.55	26.71		27.95	27.77	22.26
2017	37.93	39.71	32.23		21.34	19.85	16.03		28.92	27.19	22.24		32.59	32.18	31.58		29.83	29.52	23.94
2018	37.28	35.53	28.46		22.56	19.97	16.15		29.04	25.04	20.24		33.35	32.22	31.90		30.10	27.58	22.17
2019	52.83	42.87	34.83		27.75	21.22	17.07		44.39	33.32	27.15		34.28	23.75	23.58		40.52	30.91	25.86
APC	4.98* (0.41-9.76)	0.99 (-0.53-2.55)	2.10 (-2.57-6.91)		6.39* (1.73-11.22)	3.92* (0.45-7.53)	2.90 (-0.75-6.62)		6.26* (0.88-11.93)	1.90 (-0.2-4.0)	2.92 (-2.14-8.27)		4.64* (0.25-7.53)	0.29 (-3.91-4.70)	1.38 (-3.44-6.43)		5.39* (0.91-10.10)	1.09 (-0.43-2.64)	2.26 (-1.85-6.53)
t	2.52	1.48	1.04		3.20	2.59	1.83		2.70	2.06	1.32		2.27	0.16	0.70		2.78	1.66	1.25
P	<0.05	0.18	0.33		<0.05	<0.05	0.10		<0.05	0.07	0.22		<0.05	0.88	0.53		<0.05	0.13	0.25

APC data is expressed as a percentage, in the form of median, 95% CI. *P<0.05.

**图2:**
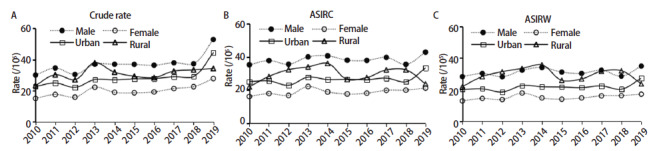
甘肃省肿瘤登记区2010-2019年肺癌发病趋势。A：粗发病率；B：ASIRC；C：ASIRW。

### 2.5 2010-2019年不同年龄组肺癌发病率变化趋势

2010-2019年，仅≥65岁组及以上年龄组肺癌发病率以年均4.15%的速度上升（P<0.05），其中男性和女性15-44岁年龄组肺癌发病率分别以年均11.18%和7.04%的速度下降，而女性和城市地区65岁及以上人群肺癌发病率分别以年均5.12%和4.88%速度增长，其余各人群各年龄组肺癌发病率变化趋势均无统计学意义（P均>0.05）。见表4、图3。

**表4:** 2010-2019年甘肃省肿瘤登记地区男性、女性、城市地区及农村地区各年龄组肺癌发病率的变化趋势（/10^5^）

Item	Age (yr)	2010	2011	2012	2013	2014	2015	2016	2017	2018	2019	APC	t	P
Total	0-14	0.00	0.00	0.00	0.00	0.88	0.00	0.00	0.00	0.00	0.31	-	-	-
	15-44	4.71	2.40	3.39	4.11	3.56	2.97	2.79	2.51	1.33	3.28	-6.01 (-13.13-1.71)	-1.80	0.11
	45-64	51.69	29.43	42.75	50.01	55.61	46.50	47.93	46.99	29.34	58.70	0.78 (-5.44-7.42)	0.28	0.79
	≥65	124.27	115.97	160.20	190.48	164.40	172.77	167.28	189.41	165.10	188.30	4.15 (0.97-7.52)	2.96	<0.05
Male	0-14	0.00	0.00	0.00	0.00	0.00	0.00	0.00	0.00	0.00	0.00	-	-	-
	15-44	5.91	5.93	3.40	4.57	3.47	3.27	2.34	2.35	1.19	3.62	-11.18 (-18.48--2.96)	-3.22	<0.05
	45-64	67.18	66.40	54.68	59.37	72.61	59.19	62.95	57.42	63.77	76.63	0.67 (-2.09-3.08)	3.52	0.56
	≥65	185.02	207.09	235.02	265.20	242.50	254.93	240.11	271.97	216.25	261.33	2.38 (-0.32-4.38)	5.16	2.03
Female	0-14	0.00	0.00	0.00	0.00	0.00	0.00	0.00	0.00	0.00	0.00	-	-	-
	15-44	3.43	4.45	3.37	3.62	3.65	2.65	3.26	2.68	1.26	2.92	-7.04 (-13.33--2.30)	-0.30	<0.05
	45-64	36.18	35.39	30.97	40.61	38.98	33.93	33.26	36.76	36.87	40.41	0.85 (-1.37-4.23)	3.12	0.88
	≥65	62.84	87.94	86.88	117.54	87.91	92.51	97.26	110.06	113.77	119.33	5.12 (1.69-10.28)	8.66	<0.05
Urban	0-14	0.00	0.00	0.00	0.00	1.25	0.00	0.00	0.00	0.00	0.35	-	-	-
	15-44	4.36	1.93	3.33	3.13	3.03	2.92	3.24	2.86	0.98	3.23	-4.83 (-14.12-5.54)	-1.102	0.30
	45-64	52.52	25.26	41.43	46.04	51.13	45.05	46.54	42.67	25.99	61.33	1.07 (-6.46-9.03)	0.30	0.77
	≥65	118.15	109.07	128.26	167.36	140.90	162.09	151.71	173.53	141.47	199.07	4.88 (1.70-8.15)	3.57	<0.05
Rural	0-14	0.00	0.00	0.00	0.00	0.00	0.00	0.00	0.00	0.00	0.25	-	-	-
	15-44	5.83	3.82	3.57	7.09	5.16	3.14	1.53	1.53	2.29	3.35	-10.28 (-19.53-0.07)	-2.29	0.05
	45-64	49.21	43.10	47.74	63.24	74.30	51.65	53.12	63.78	43.02	54.09	0.81 (-3.71-5.73)	0.41	0.69
	≥65	143.91	137.76	295.57	266.79	270.43	210.06	225.00	249.71	264.52	167.98	2.67 (-4.34-10.20)	0.85	0.42

APC data is presented as a percentage, in the form of median, 95%CI.

**图3:**
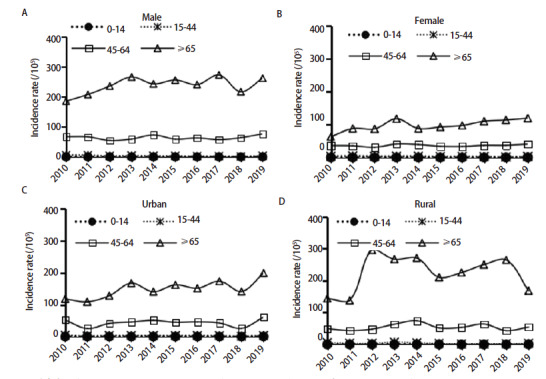
2010-2019年甘肃省肿瘤登记地区男性（A）、女性（B）、城市地区（C）及农村地区（D）各年龄组肺癌发病趋势

## 3 讨论

完整、准确、可靠的癌症发病、死亡数据对地区癌症防控策略制定发挥着非常重要的作用^[2]^。2019年甘肃省肿瘤登记地区肺癌发病的ASIRC为30.91/10万，低于2015年全国（35.96/10万）^[9]^、2016年上海市（39.76/10万）^[10]^、2017年河南省（39.20/10万）^[11]^、2017年内蒙古（38.37/10万）^[12]^、2019年河北省（31.03/10万）^[13]^水平，甘肃位于偏远的西北部地区，经济发展较其他地区相对滞后，由经济发展产生的工业废气、汽车尾气等使肺癌发病增加的环境空气污染物相对较少，这可能是甘肃省肺癌发病率均低于上述地区的主要原因。但甘肃省2019年肺癌发病率高于2018年水平^[14]^（22.03/10万），且2010-2019年甘肃省肺癌粗发病率总体呈上升趋势，上升速度较快（APC为5.39%），高于山东省（APC为4.07%）^[2]^、四川省（APC为3.75%）^[15]^、黑龙江省（APC为-5.0%）^[16]^水平，但ASIRC、ASIRW变化趋势均无统计学差异，提示甘肃省肺癌粗发病率逐年递增主要是人口老龄化引起；其次，随着甘肃经济快速发展和城镇化的进程快速推进，可能与人群接触工业环境污染、吸烟及粉尘作业等有关^[17]^，仍需要进一步研究。同时，与甘肃省通过大力推广和实施肿瘤随访登记项目、城市癌症早诊早治项目及农村癌症早诊早治项目工作，提高了人群对肺癌的知晓、自我健康监测与主动就医意识有关。

2019年登记地区肺癌发病率男性（52.83/10万）为女性（27.75/10万）的1.90倍，值得关注的是2010-2019年甘肃省肿瘤登记地区肺癌发病率年度上升值女性（APC为6.39%）是男性（APC为4.98%）的1.28倍，提示女性肺癌发病率逐年增高的趋势更值得关注。甘肃省一项基于18岁及以上人群吸烟情况的现状调查结果^[18]^显示，甘肃省18岁及以上人群吸烟率为29.80%，且女性吸烟率（1.50%）明显低于男性（68.50%），相关研究^[19]^提示中国不吸烟女性肺癌高发主要与被动吸烟及长时间暴露于通风不良的厨房吸入高温油烟有关。因此，针对男性主要以戒烟健康宣教与干预为主，女性则主要为减少二手烟及厨房高温油烟长期暴露为主，从而有效降低肺癌发病。城乡对比发现，2019年城市地区肺癌发病率（44.39/10万）为农村地区（34.28/10万）的1.31倍，且城市地区2010-2019肺癌发病率年度上升值（APC为6.26%）是农村（APC为4.64%）的1.35倍，分析其可能原因与城市地区人群接触肺癌相关危险因素频率较高，如汽车尾气、工业污染、个人不良生活习惯等^[20]^，且与城市地区人群主动就医以及城市癌症早诊早治项目的推广实施等有关。不同地区肺癌发病率亦不相同，且差异较大，农村地区肺癌发病率最高的地区是最低地区的1.49倍，但城市地区肺癌发病率最高的地区是最低地区的3.72倍，且城市地区发病率最高地区是农村地区发病率最高地区的3.80倍，城市地区发病前三位的地区均为甘肃省省会兰州市辖区，位于甘肃中东部，兰州市早在“一五”“二五”期间就是全国重点建设的工业城市之一，也是国家694个重点工业项目的规划地，工业生产引起的环境污染致使人群长期暴露于PM2.5等污染物中可能是导致兰州市辖区肺癌发病率高于其他城市地区的主要原因。

暴露于肺癌危险因素的持续时间越长，肺癌的发病风险越高^[11]^。随着年龄增长肺癌发病率呈上升趋势，40岁开始缓慢增长，55岁开始呈现明显快速上升趋势，男、女性人群分别在≥75岁组、≥80岁组达到发病高峰，总体发病趋势与全国肺癌发病特征一致^[21]^。2010-2019年65岁及以上人群肺癌发病率以年度变化4.15%的速度增长，且该年龄段女性人群、城市地区人群的增长幅度（APC分别为5.12%、4.88%）高于全人群。因此，除了将登记地区肺癌高发的40岁及以人群作为重点干预对象外，还应重点关注女性和城市地区65岁及以上年龄组人群肺癌逐年递增的问题。除对高发人群进行必要的健康教育与行为危险因素干预外，还应积极开展肺癌早期筛查，通过早期发现与诊断治疗，提高患者生活质量，降低疾病负担指数。

综上所述，甘肃省肿瘤登记地区肺癌发病率较高，特别是男性人群和城市地区居民，且2010-2019年肺癌发病率呈现逐年上升趋势。因此，需根据甘肃省肿瘤登记地区肺癌发病特征，针对肺癌高发人群的性别、年龄、城乡、地区等特征开展癌症防控的一、二级防控策略，尤其要重点关注女性人群肺癌发病率快速增长及65岁及以上人群肺癌发病逐年升高趋势的情况。针对肺癌发病的高危人群开展有针对性的健康教育与行为干预，有效降低或减少肺癌发病危险因素的暴露程度及频次，从而有效降低肺癌发病；针对肺癌高发人群开展早期筛查，通过早发现与及早治疗，提高患者生活质量及5年生存率，有效降低疾病负担，这两大举措对优化和完善甘肃省肿瘤登记地区肺癌防控工作意义重大。

**Competing interests**

The authors declare that they have no competing interests.

**Author contributions**

Liu YQ conceived and designed the study. Lu ZX analyzed the data. Ma JH, Ma JX, Zhou H, Xue JJ, Ding GH, Wang YD, Lv Z provided critical inputs on design, analysis, and interpretation of the study. All the authors had access to the data. All authors read and approved the final manuscript as submitted.
